# Investigating the Effect of Interface Temperature on Molecular Interdiffusion during Laser Transmission Welding of 3D-Printed Composite Parts

**DOI:** 10.3390/ma16186121

**Published:** 2023-09-07

**Authors:** Anh-Duc Le, André Chateau Akué Asséko, Benoît Cosson, Patricia Krawczak

**Affiliations:** IMT Nord Europe, Institut Mines Télécom, University of Lille, Centre for Materials and Processes, F-59653 Villeneuve d’Ascq, France; anh-duc.le@imt-nord-europe.fr (A.-D.L.); benoit.cosson@imt-nord-europe.fr (B.C.)

**Keywords:** molecular interdiffusion, laser transmission welding, 3D printing, composite materials, thermoplastic polymers, interface temperature, modeling, simulation

## Abstract

The present study investigated the influence of temperature on molecular interdiffusion at the interface during the laser transmission welding of 3D-printed continuous carbon-fiber-reinforced thermoplastic composites. In order to accurately measure the temperature at the weld interface, a series of thermocouples were embedded in the laser-absorbent composite part. Two different molecular interdiffusion models were implemented to calculate the degree of healing and to predict the effects of temperature on the welding process. The degree of healing and the weld line width were computed and compared with microscopy observations. The discrepancy between the two proposed numerical models was less than 6%. Both models showed good agreement with the experimental data, with an average error of 13.28% and 7.26%, respectively. The results revealed a significant correlation between the thermal history and molecular interdiffusion at the interface. Furthermore, the relationship between the welding parameters (laser beam scanning speed) and weld line width was established. The findings of this study provide a comprehensive understanding of the underlying mechanisms involved in the laser welding of 3D-printed composites and offer insights to optimize the welding process for enhanced weld quality and superior mechanical properties in the final product.

## 1. Introduction

Laser transmission welding (LTW) presents numerous advantages in welding thermoplastic polymers and composites compared to alternative conventional techniques such as ultrasonic or friction welding. LTW offers precision, flexibility, a limited heat-affected zone, effortless automation and control, and the absence of contamination [1,2]. Regarding 3D-printed continuous fiber-reinforced composites, wherein the mechanical performance remains restricted by the fiber orientation within the printing layers, and the relatively low strength of inter-layer bonding [3,4,5], LTW emerges as a prospective joining technique to form large functional assemblies. It makes it possible to produce objects in which the continuous reinforcing fibers are arranged to support multi-directional mechanical loads.

In LTW, a pair of components are involved, wherein one exhibits transparency to the laser wavelength (so-called laser-transparent part) while the other possesses absorption characteristics at the same wavelength (so-called laser-absorbent part). Prior to initiating the welding process, these components are accurately aligned. Subsequently, the laser beam energy penetrates through the transparent material and is absorbed by the absorbent material at the interface of both materials. As a consequence, the absorbent interface experiences heating, causing the materials to melt and fuse together and creating a bond between the two parts. The quality of the bond formed by a weld line is characterized by two main phenomena that define the degree of bonding: intimate contact and healing (or the molecular interdiffusion of the polymer across the bonding interface) [6]. The phenomenon of healing is intricately linked with the process of intimate contact, as it is only possible for healing to take place within regions of the interface where intimate contact is established [7]. The present study focuses on the description of the healing process assuming that complete intimate contact is already achieved at the interface. Healing can occur only when the temperature rises above the glass transition temperature ($T_{g}$) for amorphous thermoplastics or the melt temperature ($T_{m}$) for semi-crystalline thermoplastics, respectively. For the welding of fiber-reinforced thermoplastic composites, it was demonstrated in a previous work that the temperature at the interface should be well above the ($T_{g}$) of the thermoplastics to achieve optimal adhesion and complete chain entanglement [8].

The establishment of a robust bond between welded components depends on both an optimal temperature distribution within the heat-affected zone and a controlled welding time [9]. The temperature distribution and weld profile at the interface were widely determined by numerical simulations [10,11,12,13]. Validation was achieved indirectly through infrared thermography measurement at the back surface of the absorbent part [8,14]. Nevertheless, numerical simulations require knowledge of the intensity flux at the weld interface, which depends on the intensity flux distribution of the incident laser beam as well as the thickness, structure, and optical properties of the transparent part. Unfortunately, in the case of fiber-reinforced and filled composites, or semi-crystalline polymers, measuring the energy loss due to reflection and absorption within the transparent part is not trivial [15].

Recent literature reviews have highlighted the growing significance of thermoplastic composite bonding and welding processes in various industries, particularly due to the demand for lightweight, high-strength components [16,17,18]. However, challenges persist in achieving efficient and reliable welds, especially when dealing with complex geometries and various polymer materials [16,17]. The main issues related to the welding process of thermoplastic composites include (1) the need to control the heat input to avoid burning the materials; (2) the need to ensure that the materials are properly bonded together at the interface; and (3) the need to prevent the formation of voids or defects in the weld. This research endeavors to address these challenges by exploring innovative solutions to improve the thermoplastic composite welding process [19,20,21]. By examining the effect of temperature on molecular interdiffusion at the interface, this study aims to offer insights into optimizing the welding parameters, such as the laser beam scanning speed, for enhanced weld strength and quality of the final product.

Previous studies in the literature have shown that temperature plays a crucial role in controlling healing at polymer–polymer interfaces [7,22,23]. As a consequence, in this study, we aim to comprehensively investigate the effect of temperature on molecular interdiffusion at the interface in the laser transmission welding of 3D-printed continuous carbon-fiber-reinforced thermoplastic composites. The goal is to gain a better understanding of the fundamental processes that occur during the laser welding of 3D-printed thermoplastic composites. This knowledge can be used to optimize the welding process and improve the quality and mechanical properties of the final product. A unique approach in this research work is the use of an embedded set of thermocouples in the laser-absorbent composite parts, which enables an accurate temperature measurement at the weld interface. Two different non-isothermal molecular interdiffusion models are employed to predict the effects of temperature on the welding process so as to investigate the relationship between temperature and molecular interdiffusion at the interface. As mentioned above, intimate contact issues are beyond the scope of the present study; therefore, weld quality is characterized by the weld line width instead of the weld strength [24,25]. Accordingly, the relationship between the welding parameters and weld line width is analyzed.

## 2. Molecular Interdiffusion Models

**Figure 1:**
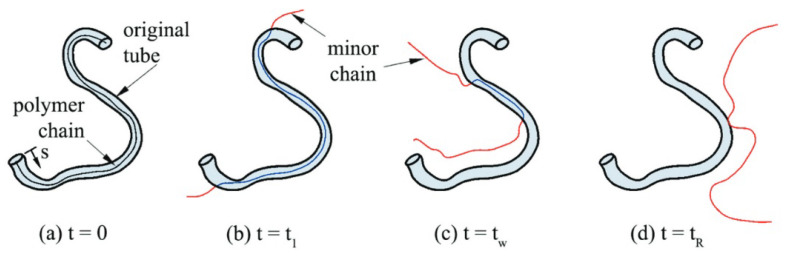
Reptation movement of a linear polymer chain [28].

In the case of isothermal conditions, the reptation theory introduced by De Gennes [26] and Doi and Edwards [27], which models the motion of individual linear polymer chains within the amorphous bulk, is frequently employed to describe the molecular interdiffusion (or healing) process. In the model, a polymer chain of length *L* is considered to be confined in an imaginary tube, which is defined by the constraints of neighboring polymer chains via entanglements. This imaginary tube restricts the movement of the chain exclusively along its curvilinear length. At the beginning of the process, t=0, the chain (i.e., the thin solid line in Figure 1) is totally encompassed by the original tube. After a period of time, $t=t_{1}$, the end of the chain, which has more mobility compared to the center of mass of the chain, escapes from the original tube, forming “minor chains” of length *l*. As time evolves, an increasing proportion of the chain leaves the original tube. During this process, *l* increases with time until it reaches (l=L) at the reptation time $t_{R}$ (see Figure 1).

**Figure 2:**
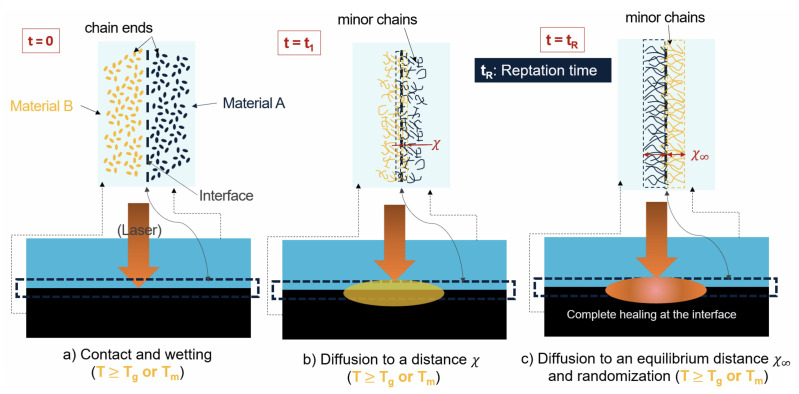
Interdiffusion of minor chains across a polymer–polymer interface during the laser transmission welding process.

The polymer molecular interdiffusion at the interface between two miscible thermoplastics during the LTW process is depicted schematically in Figure 2. At the origin (t=0), all the minor chains have zero length as denoted by the dots. At $t=t_{1}$, the reptation of the minor chains initiates, and some of the minor chains cross the interface by an average distance denoted as χ. Finally, when the time is close to the reptation time or relaxation time $t=t_{R}$, the interdiffusion is fully developed and chain entanglements are achieved (i.e., $\chi =\chi_{\infty }$, and l=L) [22,29]. For a time longer than the reptation time, the interface disappears, and the properties of the virgin bulk material are reached [30]. The bond strength, σ, is characterized by the average interpenetration distance of the chains across the interface χ, which varies as the square root of the minor chain length as $\chi \approx \sqrt{l}$. By using Einstein’s diffusion equation (Equation (1)), it is possible to show that healing is related to time to a power of 1/4 [31]:

(1) $$\langle l^{2}\rangle =2Dt\Rightarrow \chi \approx \sqrt{l}={\langle l^{2}\rangle }^{1/4}\propto t^{1/4}$$

where $\langle l^{2}\rangle$ is the quadratic distance of diffusion, and *D* is the self-diffusion coefficient. Finally, the degree of healing, $D_{h}$, is defined as the ratio of the instantaneous interfacial bond strength to the ultimate bond strength as [7,26]

(2) $$D_{h}=\frac{\sigma }{\sigma_{\infty }}=\frac{\chi }{\chi_{\infty }}={\left(\frac{l}{L}\right)}^{1/2}={\left(\frac{t}{t_{R}}\right)}^{1/4}$$

Regarding the LTW process, the temperature field exhibits temporal variation. Furthermore, the temperature distribution within a weld line is uneven. Bastien and Gillespie [23] were the pioneers in introducing the non-isothermal healing model for the fusion bonding of amorphous polymers. In this model, the thermal history can be divided into *n* time intervals, in which the average temperature $T_{i}^{*}$, between times $t_{i}$ and $t_{i+1}$, is assumed constant. In this manner, the application of the healing theory is feasible during every individual isothermal increment. Bastien and Gillespie [23] proposed two mathematical models for the prediction of the bond strength, which were based on the minor chain length criteria and the average interpenetration distance criteria. Szuchács et al. [28] have recently demonstrated that the first model (Equation (3)) is more appropriate compared to their experimental data. Therefore, this model (hereafter “Model 1”) is employed in the framework of this research work:

(3) $$D_{h}=\frac{\sigma }{\sigma_{\infty }}={\left(\frac{l}{L}\right)}^{1/2}={\left[\sum_{i=0}^{n}\left(\frac{t_{i+1}^{1/2}-t_{i}^{1/2}}{t_{R}{\left(T_{i}^{*}\right)}^{1/2}}\right)\right]}^{1/2}$$

The bond strength can also be assessed by calculating the quadratic distance of diffusion according to the formulation depicted in Equation (1). On the basis of the work of Doi and Edwards [32], Graessley [33] pointed out that the self-diffusion coefficient may be calculated from the measurable viscoelastic quantities of the polymer as in Equation (4):

(4) $$D=\frac{G_{N}^{0}}{135}{\left(\frac{\rho RT}{G_{N}^{0}}\right)}^{2}\left(\frac{\langle r^{2}\rangle }{M_{w}}\right)\frac{M_{c}\left(T\right)}{M_{w}^{2}\eta_{0,M_{c}\left(T\right)}}$$

where $G_{N}^{0}$ is the plateau modulus, ρ is the polymer density, *R* is the universal gas constant, *T* is the absolute temperature, $\langle r^{2}\rangle$ is the unperturbed mean square end-to-end distance of the chain, $M_{w}$ is the molecular weight, $M_{c}\left(T\right)$ is the critical molecular weight at the temperature *T*, and $\eta_{0,M_{c}\left(T\right)}$ is the zero-shear viscosity at the critical molecular weight and the temperature *T*.

The $G_{N}^{0}$ modulus can be deduced from the master curve of the considered polymer, which can be recorded through dynamic rheological measurements. The critical molecular weight $M_{c}\left(T\right)$ is linked to the molecular weight of entanglements $M_{e}\left(T\right)$ by Equation (5) [34]:

(5) $$M_{c}\left(T\right)=2M_{e}\left(T\right)\;\mathrm{with}\;M_{e}\left(T\right)=\frac{\rho RT}{G_{N}^{0}}$$

The unperturbed mean square end-to-end distance of the chain is difficult to determine experimentally. It was stated that the ratio between this distance and the molar mass $M_{w}$ is a constant [35]. In the case of the thermoplastic polymer used in the present study, namely poly-ethylene terephthalate glycol-modified (PETG), this ratio is fixed as [35]

(6) $$\frac{\langle r^{2}\rangle }{M_{w}}=0.845{\overset{o}{A}}^{2}\mathrm{mol}\cdot g^{-1}$$

Note that the value associated with PET was used for this calculation due to the overall similarities and a lack of prior literature covering PETG diffusion dynamics.

The relationship between the zero-shear viscosity $\eta_{0}$ and the molecular weight $M_{w}$ can be expressed by the following equation [33]:

(7) $$\eta_{0}=k_{H}M_{w}^{3.4}$$

where $k_{H}$ is a material constant. Utilizing the values for the known zero-shear viscosity and molecular weight in Ref. [36], it is possible to solve for $k_{H}$. Subsequently, a molecular weight of $M_{w}=$ 28,126 g·mol^−1^ was estimated for the PETG used in the present study.

Since the numerical value for $\eta_{0,M_{c}\left(T\right)}$ was not available, we used the following relationship (Equation (8)) for the calculations, as also carried out by Kim and Han [37]:

(8) $$\frac{\eta_{0,M_{c}\left(T\right)}}{\eta_{0,M_{w}\left(T\right)}}={\left(\frac{M_{c}\left(T\right)}{M_{w}\left(T\right)}\right)}^{3.4}$$

As suggested in [8,38,39], complete interdiffusion can only be achieved when the quadratic distance of diffusion is greater than the mean square end-to-end distance ($\langle r^{2}\rangle =2.38\times 10^{-16}$ m^2^). Therefore, the degree of healing ($D_{h}$) can be calculated by the following equation (hereafter “Model 2”):

(9) $$D_{h}=\frac{\sigma }{\sigma_{\infty }}={\left(\frac{\langle l^{2}\rangle }{\langle r^{2}\rangle }\right)}^{1/4}={\left[\sum_{i=0}^{n}\frac{2D\left(T_{i}^{*}\right)\left(t_{i+1}-t_{i}\right)}{\langle r^{2}\rangle }\right]}^{1/4}$$

## 3. Experimentals

### 3.1. Materials

The 3D-printed specimens used for the welding experiments were made of poly-ethylene terephthalate glycol-modified (PETG) and continuous carbon-fiber-reinforced PETG composites (CCFPC). A natural transparent and a black-pigmented PETGs were chosen for the semi-transparent and the absorbent parts, respectively. The materials were supplied by Polymaker™ in the form of 1.75 mm round filaments. According to the supplier, the PETG polymer had a density of 1.25 g·cm^−3^ and a glass transition temperature of 81 °C. The nozzle temperature was recommended in the range of (230–260 °C). The composite carbon fiber (CCF) filament of 0.35 mm diameter was supplied by Anisoprint™, which comprised 60% in volume of carbon fibers pre-impregnated with an epoxy thermosetting resin to provide good adhesion with the thermoplastic matrix.

### 3.2. 3D Printing of Continuous Carbon-Fiber-Reinforced Thermoplastic Composites

**Figure 3:**
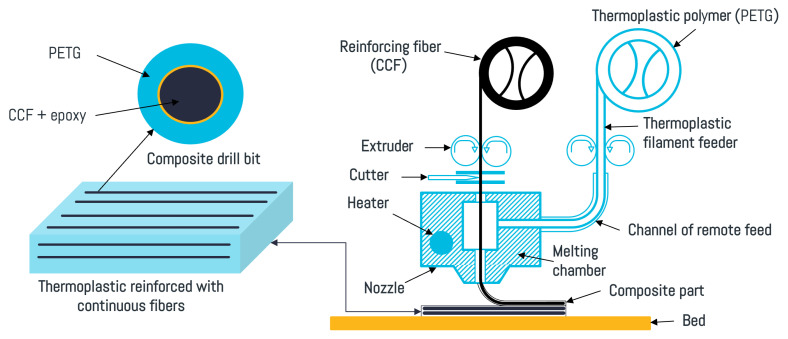
Schematic view of the Anisoprint™ printer used to manufacture the continuous carbon-fiber-reinforced PETG composites.

The CCFPCs were printed using a Composer A4 Desktop 3D Printer from Anisoprint™. The printing process was based on the composite fiber co-extrusion technology. In this printing technology, both the polymer and the CCF filaments are fed from two different spools to a common printing head (see Figure 3). In this way, the molten polymer wets the CCF in the nozzle prior to printing. The Aura™ software was used to slice the 3D CAD models and assign the processing parameters to the G-codes. The 3D CAD models used to manufacture the welding specimens had a rectangular bar shape of 80 × 20 × 2 mm^3^. The nozzle temperature was $T_{n}$ = 260 °C, and the bed temperature was $T_{b}$ = 75 °C. The printing speed was 10 mm·s^−1^ and the layer thickness was 0.2 mm for the plastic nozzle and 0.4 mm for the composite nozzle. The fill density was 100%, and the fiber fill type was set to line with an angle of 0°.

### 3.3. Laser Transmission Welding of Composites

**Figure 4:**
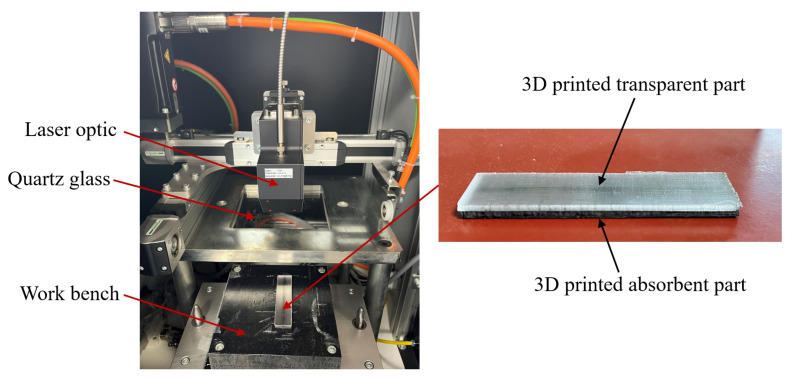
Laser transmission welding of 3D-printed components.

The LTW experiments were conducted using two types of 3D-printed components: natural PETG-T, which is semi-transparent at the laser wavelength, and CCFPC-A consisting of black-pigmented PETG and CCF, which is absorbent at the same wavelength. The process setup is illustrated in Figure 4. The laser welding machine (LEISTER NOVOLAS™) was equipped with a diode laser transported by an optic fiber. The maximum output power was 46 W and the laser wavelength was 0.940 μm. The semi-transparent PETG-T part was placed above the CCFPC-A absorbent part on a workbench and fixed with a transparent tape. The two components to be welded were located between a transparent cover and the workbench, which was displaced by a pneumatic actuator. The quartz glass used as the transparent cover allowed 95% of laser energy transmitted through it at the laser wavelength. For this application, the used laser power was set at 10 W in the welding system, and the clamping pressure was 6 bar in the pneumatic system. Two sequences of scan lines were run at different scanning speeds (i.e., *v* = 1.36 mm·s^−1^, *v* = 2.72 mm·s^−1^, and *v* = 4.08 mm·s^−1^).

### 3.4. Temperature Measurement at the Weld Interface

**Figure 5:**
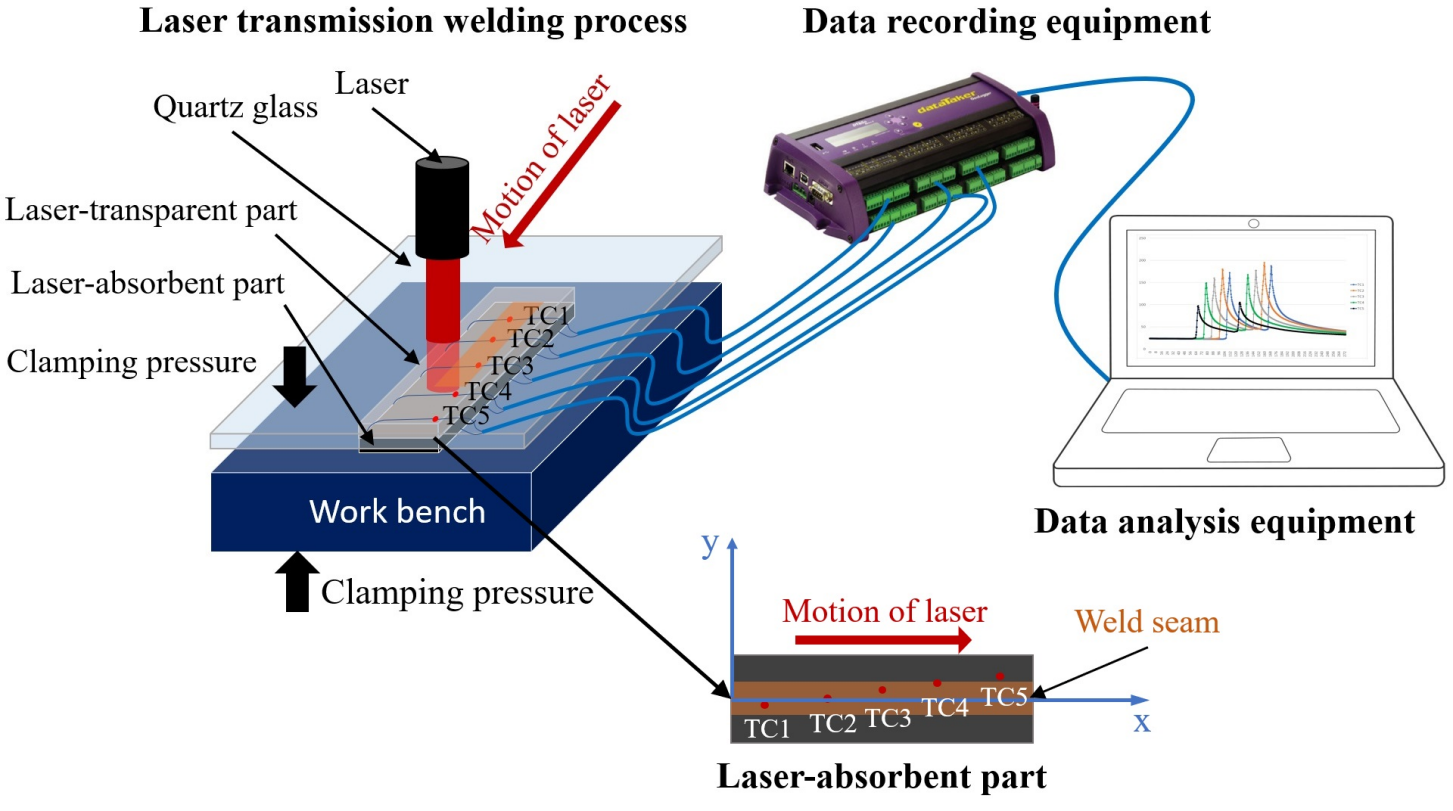
Illustration of laser transmission welding process and temperature measurement at the weld interface. TC stands for thermocouple.

The monitoring of the thermal evolution occurring at the interface during the LTW procedure was carried out using 5 k-type thermocouples with a wire diameter of 0.12 mm. The thermocouples were strategically embedded into designated locations (as specified in Table 1) within the holes of the absorbent component, with a penetration depth slightly exceeding the thermocouple junction size from the top surface. Once the thermocouples were appropriately positioned, the exact locations of the thermocouple junctions were reassessed. The measurements were conducted with accuracy of 0.01 mm (in Table 1). The samples were prepared with wide welding in order to create perfect contact. Following the initial trial, the thermocouples were completely wet in the thermoplastic material. Subsequently, repeated experiments were conducted on the same sample to measure the temperature field. The data were recorded using the Datataker DT85M data logger device (as illustrated in Figure 5). The frame rate for capturing the temperature at the interface was 120 Hz, and the measurement uncertainty was ±2.5 °C.

### 3.5. Rheological Characterization of the Thermoplastic

The rheological properties of PETG were investigated using an Anton Paar Physica MCR 301 rotational rheometer. The measurements were conducted using a parallel-plate geometry with a diameter of 35 mm at a 1 mm gap. Nitrogen was employed as a protective gas to prevent polymer degradation. The rheological measurement specimens were 3D printed, employing identical parameters to those employed for the production of the PETG-T parts. Frequency sweep tests were performed to determine the storage modulus $G^{\prime }$, the loss modulus $G^{\prime \prime }$ and the complex viscosity $\eta^{*}$ at different temperatures: 130 °C, 150 °C, 170 °C, 190 °C, 210 °C and 230 °C. With respect to the linear viscoelastic range, the shear strain applied was 5%. The angular frequency was varied from 0.1 rad·s^−1^ to 628 rad·s^−1^.

## 4. Results and Discussion

### 4.1. Temperature Measurement

**Figure 6:**
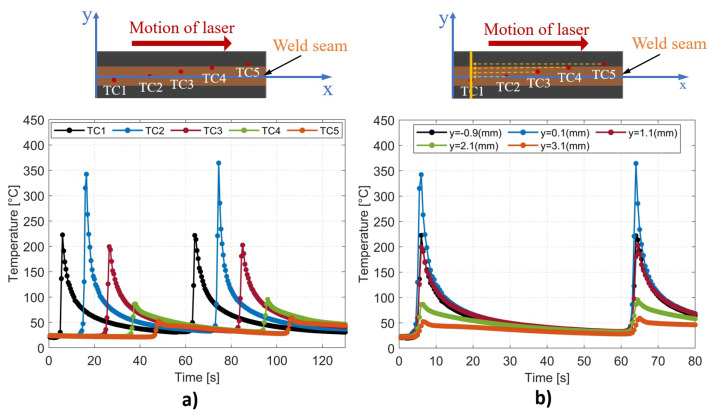
(**a**) Experimental interface temperatures measured at five different positions with five thermocouples. (**b**) Thermal history at five different positions on a perpendicular line passing through the thermocouple TC1 using shifted time.

Figure 6a illustrates the temporal variation in the interface temperature recorded by five thermocouples following two successive passages of the laser beam. The highest temperature was recorded by TC2 positioned at the center of the laser spot, while the temperatures gradually decreased with increasing distance from the central line of the part. The cooling was instantaneous following the passage of the laser on each thermocouple via thermal conduction and natural convection. In comparison with our previous research conducted on thermoplastic PLA [40], the cooling rate was accelerated as a result of the higher thermal conductivity of the CCFPC compared to the pure thermoplastic PLA.

For the LTW process with a constant scanning speed of the laser, except for the two end sections of the part subjected to boundary effects, the thermal history of all perpendicular lines to the weld seam was considered to be identical. Accordingly, the temperature measurement by five thermocouples at different positions along the weld seam could be used to describe the thermal history occurring on a single line perpendicular to the weld seam, as shown in Figure 6b. For this purpose, a shift factor was applied to the time, which corresponded to the duration taken by the laser to travel a distance equal to the spatial gap between the thermocouple position and the specified line.

**Figure 7:**
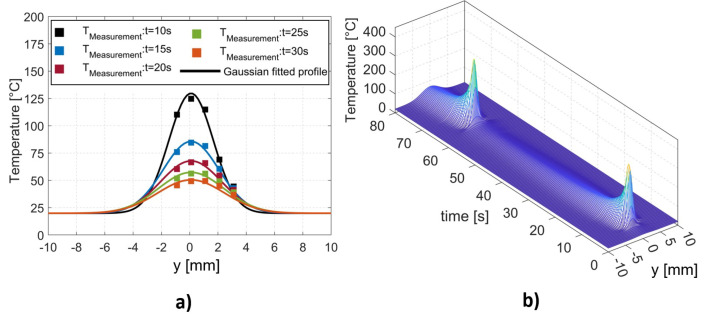
(**a**) Gaussian–fitted profile for temperature on a line perpendicular to the weld seam at different moments. (**b**) Full thermal history of a line perpendicular to the weld seam during the laser transmission welding process.

The instantaneous temperature profile along a perpendicular line to the weld seam has been demonstrated to have a Gaussian shape [40,41]. Based on the outcomes presented above, it is evident that employing a Gaussian-fitted model makes it possible to generate a temperature profile for every temporal instance (see Figure 7a). Finally, the full thermal history of the specified line was computationally reconstructed, as shown in Figure 7b. These temperature data were then used for the calculation of the degree of healing.

### 4.2. Rheological Characterization

**Figure 8:**
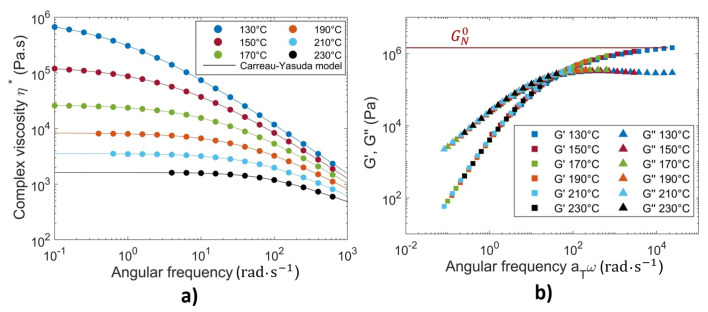
(**a**) Complex viscosity (circle symbol) versus angular frequency. (**b**) Time–temperature superposition master curves of storage modulus $G^{\prime }$ (square symbol) and loss modulus $G^{\prime \prime }$ (triangle symbol) at 170 °C for PETG.

Figure 8a plots the results of the complex viscosity versus the angular frequency, obtained from dynamic frequency sweep tests at temperatures ranging from 130 °C to 230 °C. The variation in the viscosity can be well fitted by the Carreau–Yasuda model (Equation (10)), where the zero-shear viscosity ($\eta_{0}$) and the relaxation time (λ) are produced (as shown in the Table 2)

(10) $$\eta =\eta_{0}{\left[1+{\left(\lambda \omega \right)}^{a}\right]}^{\frac{n-1}{a}}$$

where *a* stands for the width of the transition range between zero-shear viscosity and the power law regime, and *n* is the power law exponent. The Carreau–Yasuda regression parameters giving the best fits to the experimental data are listed in Table 2.

The master curves were constructed by time–temperature superposition (TTS) from dynamic frequency sweep tests obtained at different temperatures. Figure 8b shows the master curves of the storage modulus $G^{\prime }$ and the loss modulus $G^{\prime \prime }$ as functions of the angular frequency obtained at the reference temperature of 170 °C. The results demonstrate the linear viscoelastic characteristics of the PETG material. As illustrated in Figure 8b, the plateau zone of the $G^{\prime }$ curve is clearly visible when the frequency is above 10${}^{4}$ rad·s^−1^ within the superposed angular frequency range. The plateau modulus $G_{N}^{0}$ was determined as equal to the storage modulus $G^{\prime }$ at the angular frequency, where $G^{\prime \prime }$ reaches a minimum in the plateau zone [42].

**Figure 9:**
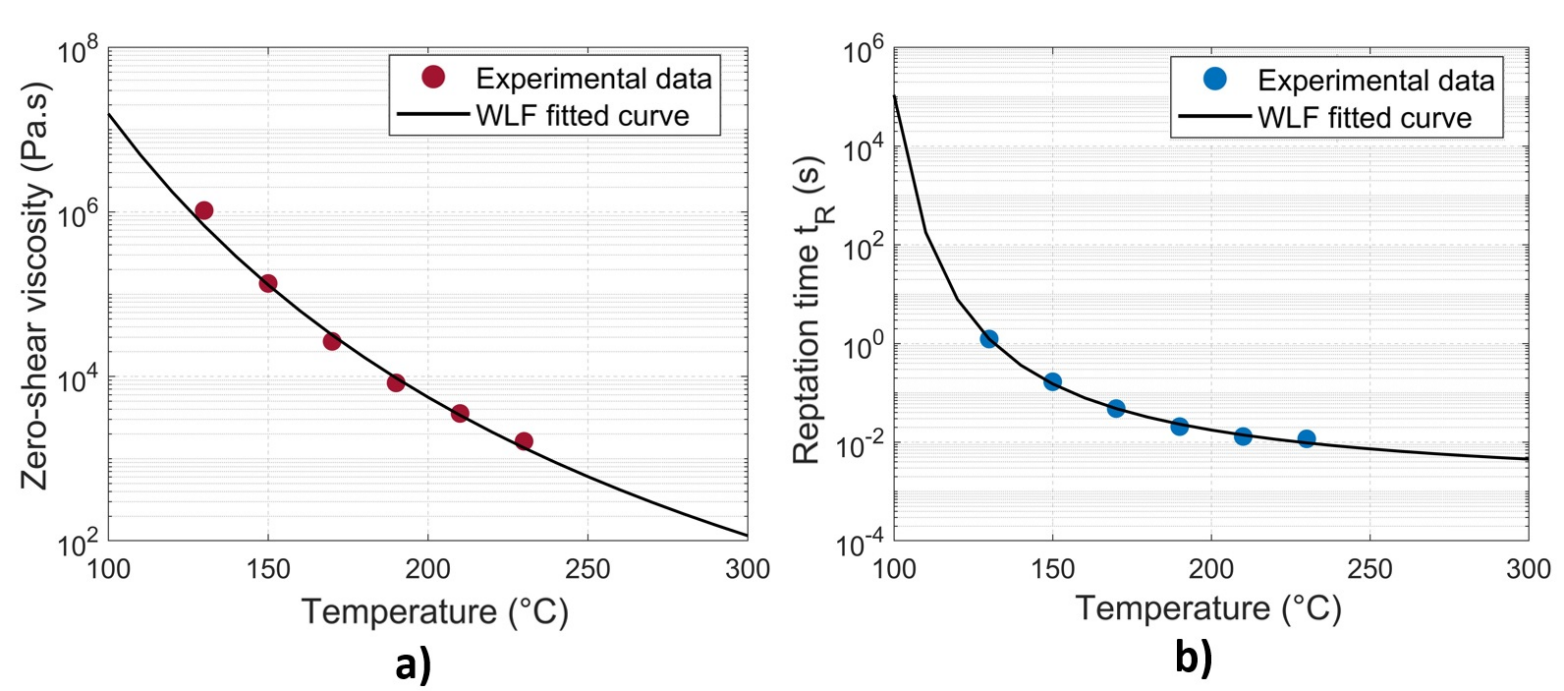
(**a**) Zero-shear viscosity versus temperature for PETG. (**b**) Reptation time versus temperature for PETG at $T_{ref}$ = 170 °C.

The widely accepted Williams–Landel–Ferry (WLF) model was employed to characterize the temperature-dependent behavior of the zero–shear viscosity as in Equation (11):

(11) $$\eta_{0}=D_{1}\exp\left[\frac{-A_{1}\left(T-T_{g}\right)}{A_{2}+\left(T-T_{g}\right)}\right]$$

where $D_{1}$, $A_{1}$, $A_{2}$ are data-fitted parameters. The reptation time ($t_{R}$) of an amorphous material was considered to be equivalent to the relaxation time (λ) [6,28,29], which was derived from viscosity measurements (see Table 2). It obeys also a WLF law as in Equation (12):

(12) $$t_{R}\left(T\right)=a_{T}t_{R}\left(T_{ref}\right)\;\mathrm{with}\;\log a_{T}=\frac{-C_{1}\left(T-T_{ref}\right)}{C_{2}+\left(T-T_{ref}\right)}$$

where $a_{T}$ is the shift factor, and $C_{1}$ and $C_{2}$ are constants. The data-fitted parameters for WLF models of the zero-shear viscosity ($\eta_{0}$) and the reptation time ($t_{R}$) at $T_{ref}$ = 170 °C are listed in Table 3. The semilog plot of the experimentally obtained data and the corresponding WLF fitted curves are given in Figure 9.

### 4.3. Effects of Temperature on Molecular Interdiffusion at the Interface

**Figure 10:**
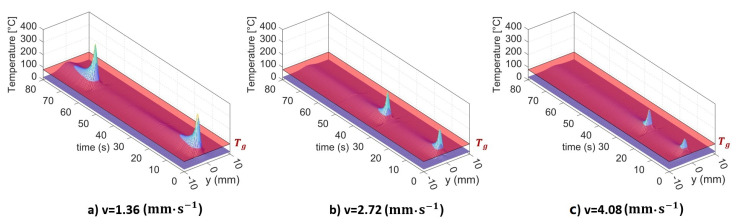
Thermal history of a line perpendicular to the weld seam for different scanning speeds of the laser beam.

In order to investigate the effects of temperature on molecular interdiffusion at the interface, LTW trials with different scanning speeds were carried out: *v* = 1.36 mm·s^−1^, *v* = 2.72 mm·s^−1^, and *v* = 4.08 mm·s^−1^. Temperature measurements were performed and the thermal histories of a line perpendicular to the weld seam were established, as depicted in Figure 10. In this figure, the glass transition temperature ($T_{g}$) is represented by the semi-transparent red plane. It appears that variations in the scanning speed yield disparities in the thermal history of the specified line. Decreasing the scanning speed leads to an enlarged weld zone where the temperature exceeds the glass transition temperature $T_{g}$, as well as an extended duration during which the temperature remains above $T_{g}$. These obtained data serve as essential variables for the calculation of the degree of healing along the specified line.

**Figure 11:**
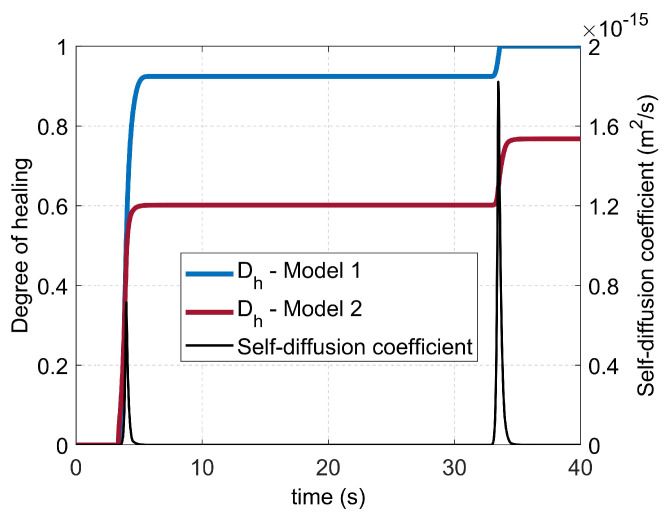
Evolution of self-diffusion coefficient (*D*) and degree of healing ($D_{h}$) versus time at *y* = 0.8 mm for scanning speed *v* = 2.72 mm·s^−1^.

Two different non-isothermal models, Equations (3) and (9), respectively, were implemented numerically to predict the degree of healing as a function of time. The calculations were performed for each node along the perpendicular line. It is important to note that the healing mechanism exclusively occurs when the interface temperature exceeds the glass transition temperature $T_{g}$. Therefore, for any time increment, if the temperature is below $T_{g}$, molecular interdiffusion will not take place at this node and the degree of healing will not increase. Once a degree of healing of unity is achieved, the state of full healing is preserved and remains unaffected by subsequent fluctuations in temperature (see Figure 11). For Model 1 (Equation (3)), only the reptation time ($t_{R}$) at given temperatures was needed for the calculation; it was calculated with the WLF equation (Equation (12) and Figure 9b). For Model 2 (Equation (9)), all the data for the self-diffusion coefficient calculation of the used PETG are reported in Table 4. The zero-shear viscosity was calculated using the WLF model (Equation (11) and Figure 9a). Then, the zero-shear viscosity at the critical molecular weight and the temperature *T* ($\eta_{0,M_{c}\left(T\right)}$) was computed according to Equation (8). The evolution of the self-diffusion coefficient (*D*) and the degree of healing ($D_{h}$) versus time, calculated at the location *y* = 0.8 mm for the case v=2.72 mm·s^−1^, is illustrated in Figure 11.

**Figure 12:**
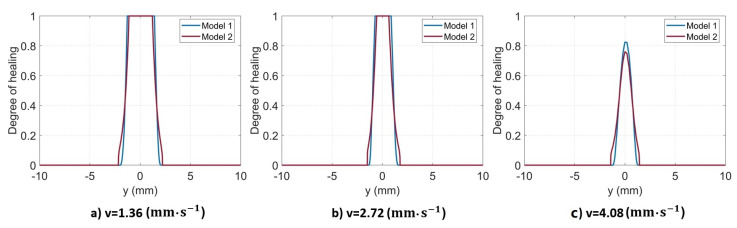
Computed degree of healing along a line perpendicular to the weld seam using two different models.

Figure 12 plots the calculated results obtained from the two non-isothermal models. It is obvious that there is a direct correlation between the decreased scanning speed and the increased width of the weld seam. This finding can be explained by the fact that the molecular interdiffusion is promoted by the elevated temperature at the interface and the longer welding duration. As a result, the lower the scanning speed is, the larger the weld line width is. It is evident that both models exhibit small disparities in their predictions of the degree of healing ($D_{h}$). For the first two cases with higher interface temperatures, complete healing is achieved at the center of the weld seam, wherein the degree of healing reaches unity ($D_{h}=1$). Conversely, at the highest scanning speed, both models predict that complete healing has not yet been achieved within the weld seam.

**Figure 13:**
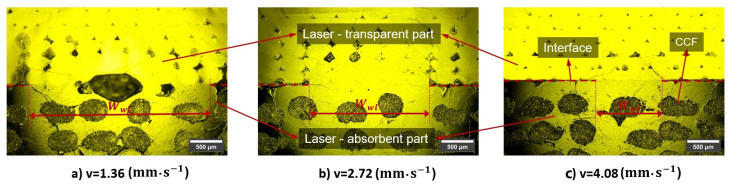
Computed weld line width using optical microscopy images.

**Figure 14:**
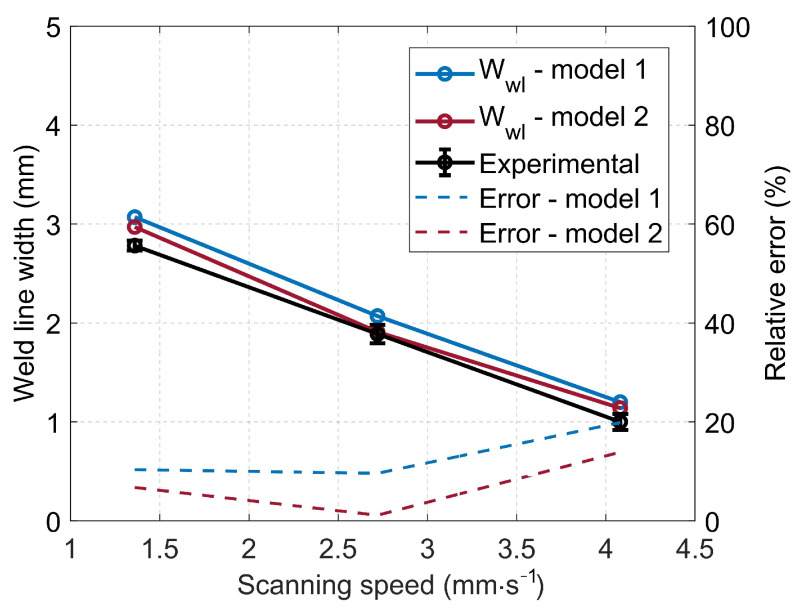
Prediction of weld line width as a function of laser scanning speed.

To assess the interdiffusion models, a comparative analysis was conducted between the predicted and experimental weld line widths. Optical microscopy examination of the cross-sectional assembled parts was performed to obtain information on the weld line width ($W_{wl}$), as depicted in Figure 13. For the visualization of the weld zone in the optical microscopy images, the degree of healing ($D_{h}$) at the edge of the weld seam should be relatively consistent. As reported by [24], $D_{h}$ varies between 0 and 1 at the edge of the weld seam. For consistency, an average value of $D_{h}=0.5$ was used in this study to predict the theoretical weld line widths for both numerical models. Figure 14 shows a comparison of the predicted and measured weld line widths as a function of the laser scanning speed. It appears that both models are reasonably efficient in predicting the weld line width and that the discrepancy between them is relatively small (i.e., less than 6%). The average errors of the two models compared to the experimental data are 13.28% and 7.26%, respectively. Generally, Model 2 demonstrates a commendable predictive capability, which results in a better correlation with the experimental data across all investigated scenarios.

In Figure 13a, the sample was welded with excessively high line energy (i.e., the ratio of the laser power to the scanning speed); thereby, the interface was overheated, yielding thermal degradation and pores in the joint, as also reported in Ref. [9]. This experimental phenomenon could not be predicted with the proposed numerical methods.

## 5. Conclusions

A novel approach has been proposed for the comprehensive investigation of the effect of temperature on molecular interdiffusion during the laser transmission welding of 3D-printed continuous carbon-fiber-reinforced thermoplastic composites. The novelty of the approach lies in the implementation of an embedded set of thermocouples within the laser-absorbent component, which permits an accurate temperature measurement at the weld interface. Using the data obtained from five thermocouples embedded at different positions, the thermal history of a line perpendicular to the weld seam was reconstructed. Two non-isothermal molecular interdiffusion models were proposed and employed to predict the degree of healing across the specified line. The accuracy and reliability of the molecular interdiffusion models were assessed by comparing the weld line widths predicted by numerical models with the measurements derived from optical microscopy of the cross-sectional assembled parts. The results revealed a significant correlation between the thermal history and molecular interdiffusion at the weld interface. Moreover, the investigation highlighted the relationship between the welding parameters (i.e., the scanning speed) and weld line width. Both the proposed numerical models predicted the weld line width fairly accurately and produced a good correlation with the experimental data across all examined cases. The discrepancy between the two numerical modes was less than 6% and the average errors compared to the experimental data were 13.28% and 7.26%, respectively.

By understanding the mechanisms involved in the laser welding of 3D-printed composites, the findings can be utilized to optimize the welding process, resulting in improved weld quality and enhanced mechanical properties in the final product.

## Figures and Tables

**Table 1:** Coordinate positions of the thermocouples.

Thermocouple Number	TC1	TC2	TC3	TC4	TC5
x (mm)	10.00	24.00	37.95	52.80	67.00
y (mm)	−0.90	0.10	1.10	2.10	3.10

**Table 2:** Carreau–Yasuda regression parameters.

Temperature (°C)	$\eta_{0}$ (Pa.s)	λ (s)	*a*	*n*
130	1,043,395	1.2363	0.56	0.09
150	135,215	0.1682	0.60	0.10
170	26,704	0.0481	0.71	0.18
190	8346	0.0207	0.81	0.26
210	3548	0.0131	0.90	0.35
230	1627	0.0117	1.20	0.52

**Table 3:** WLF fitted parameters for zero-shear viscosity ($\eta_{0}$) and the reptation time ($t_{R}$).

Parameter	$D_{1}$ (Pa.s)	$A_{1}$	$A_{2}$ (K)	$C_{1}$	$C_{2}$ (K)
Value	2.0115 $\times \;10^{8}$	25.65	171.94	1.7251	89

**Table 4:** Data for self-diffusion coefficient calculation of PETG.

Parameter	$G_{N}^{0}$ (Pa)	ρ (g·m^−3^)	*R* (J·(mol·K)${}^{-1}$)	$M_{w}$ (g·mol^−1^)	$\langle r^{2}\rangle$ (m^2^)
Value	1,298,300	1.25 $\times \;10^{6}$	8.314	28,126	2.38 $\times \;10^{-16}$

## Data Availability

The data presented in this study are available on request from the corresponding author.
